# Enacting Metaphors in Systemic Collaborative Therapy

**DOI:** 10.3389/fpsyg.2022.867235

**Published:** 2022-05-04

**Authors:** Zuzanna Rucińska, Thomas Fondelli

**Affiliations:** ^1^Centre for Philosophical Psychology, Department of Philosophy, University of Antwerp, Antwerp, Belgium; ^2^Interactie Academie, Antwerp, Belgium

**Keywords:** systemic therapy, enactivism, metaphors, embodiment, dialog

## Abstract

What makes metaphors good therapeutic tools? In this paper, we provide an answer to this question by analyzing how metaphors work in systemic collaborative therapeutic practices. We look at the recent embodied, enactive and ecological proposals to metaphors, and provide our own, dialogical-enactive account, whereby metaphors are tools for enacting change in therapeutic dialogs. We highlight the role of enacting metaphors in therapy, which is concerned with how one uses the metaphors in shared process of communication. Our answer is that metaphors serve as good tools for connecting to action words, through which the client’s embodiment and agency can be explored. To illustrate our view, we analyze two examples of enacting metaphors in therapeutic engagements with adolescents. Our enactive proposal to metaphors is different from others as it does not rely on engaging in explicit performances but stays within a linguistic dialog. We take metaphoric engagement as an act of participatory sense-making, unfolding in the interaction. This insight stems from enactive ways of thinking about language as a process accomplished by embodied agents in interaction, and seeing talking also as a form of doing.

## Introduction

Metaphors are used often in many therapies, whether to explore the clients’ problems, generate new insights, or to introduce a new outlook on their lives (see, e.g., Tay, 2013; Stoddard and Afari, 2014; Stilwell et al., 2021). Metaphors have also been an important tool for systemic collaborative therapists, who use it to explore their client’s experiences and facilitate change (Anderson, 1997; Antoine, 2017).

But what makes metaphors good therapeutic tools? Classical conceptual theory of metaphor (Lakoff and Johnson, 1980, 1999) proposes that metaphoric thinking is based on conscious violation of established categories and mapping from source to target domains. Following this linguistic framework, metaphors allow for a “transfer of meaning,” or thinking through one thing in terms of another. In this paper, we propose to answer this question from a different perspective, one of Embodied and Enactive Cognition (EEC) and systemic collaborative therapy. Embodied and enactive accounts of metaphors (Gallagher and Lindgren, 2015; Stilwell et al., 2021) ground metaphoric thinking in bodily and motoric processes, and propose that metaphors are enacted in ongoing physical interactions. The enactive approach in particular proposes a new way of thinking about metaphors. It steps away from the view of metaphors as linguistic symbols that only “rename” one thing as another. Instead, it suggests that metaphors can emerge in participatory sense-making activities (De Jaegher and Di Paolo, 2007), act as means for further engagement, and should be treated as useful tools for interaction and communication (Rucińska et al., 2021). Furthermore, we highlight the idea that we can enact metaphors. Enacting metaphors is concerned with how one uses the words in shared process of communication. We will show that, importantly, it is not always the metaphor on its own that achieves the change in therapeutic context; it is the skillful use of the metaphor by the therapist that allows the metaphoric dialog to bring about new meanings and action possibilities. To contextualize our insights, we will show examples from systemic collaborative therapeutic practice. Systemic collaborative approach to psychotherapy already embraces the idea that psychotherapy is about creating a context in which therapist and his/her clients, in conversation, explore and co-construct new meanings together (Anderson, 1997; Rucińska and Reijmers, 2015).

Our enactive approach to metaphors is different from other recently available ones (Gallagher and Lindgren, 2015; Stilwell et al., 2021), which mostly focus on the bodily movements and performances involved in enacting metaphors, leaving an explanatory gap for speaking about enacting metaphors in dialog alone. We argue that metaphors can be enacted not just in play and in movement, but also in dialog, as talking is also a form of doing. As a result, we can enact different possibilities and find new senses or meanings in therapeutic conversation. In fact, a phrase need not even be a standing metaphor for one to enact it as such in dialog.

This paper proceeds as follows. In section “Systemic Collaborative Therapy Meets Embodied and Enactive Cognition,” we introduce main tenets of systemic collaborative therapy and show how it fits with EEC. In section “Metaphors in Psychotherapy and Systemic Collaborative Practices,” we discuss how metaphors are used in systemic collaborative practice, and in section “Embodied, Enactive and Ecological Metaphors,” we clarify how EEC views metaphors. In section “How Do Metaphors Work?,” we analyze how embodied, enacted, and ecological metaphors are supposed to work. In section “Enacting Metaphors in Dialog: Examples From the Therapeutic Practice”, we illustrate what it means to enact metaphors in dialog with two examples from systemic therapeutic practice. In section “What Makes Metaphors Good Therapeutic Tools? An Analysis”, we provide an analysis of what, in our view, makes metaphors good therapeutic tools. The gist of our proposal is that metaphors work best in therapeutic dialog when they are matched with action words. Action words allow the therapist to change the meaning of client-generated metaphors and lay the building blocks for his/her client to increasingly experience a sense of agency. Thus, the transformative character of metaphors in therapeutic encounters resides in their use, in so far as they can be linked to action words, enhancing, in this way, the agency of the client and promoting the co-construction of shared meanings (or participatory sense-making).^1^ Section “Conclusion and Follow-Up” concludes the paper with insights about applicability of our proposal to other therapeutic contexts and provides insights for future work.

## Systemic Collaborative Therapy Meets Embodied and Enactive Cognition

A systemic approach to psychotherapy proposes that people are nodes in a complex network of relationships that affect them in their thinking, feeling, and doing; it focuses on the mutual and complex influences between the client and the networks in which he or she is interwoven (Bertrando, 2007). Contrary to what is often believed, systemic approaches to psychotherapy are not limited to working with couples or families: the systemic paradigm in which people are understood as nodes in networks of relationships allows working with individuals, couples, families, and groups (Boscolo and Betrando, 1996; Viou and Georgaca, 2019). Systemic collaborative therapy, as developed among others by Harlene Anderson, is one type of systemic therapy that specifically focuses on developing a collaborative relationship between the therapist and the individual, and engaging in dialogs that encourage growth and change. Systemic collaborative therapy emphasizes how social systems are linguistic systems (Anderson, 1997): it is through language, both spoken and unspoken, that people give meaning to themselves, others, and the world they live in Anderson and Goolishian (1992). The meanings that people co-create are socially constructed meanings—they take shape within dialogs and interactions (Rucińska and Reijmers, 2015), that are in turn influenced by, and must be situated within, broader social and cultural discourses. In the collaborative construction of stories and meanings, the therapist and the client strive for the client to experience a “relational sense of agency” (De Mol et al., 2018), which refers to how clients can once again experience they (can) make a difference in the networks of relationships in which they live. In the dialogical space that is created between the therapist and the client, the client can re-experience how he/she appears in relational contexts in a multitude of ways, and thus develop new perspectives on him/herself, the other, and the world.

This systemic perspective, informed by postmodern thought, also requires a critical look at the position of the therapist. In some therapeutic currents, the therapist is given the position of an expert: he or she can judge what is normal or pathological, what causes pathology, and what is needed to become healthy again. From a postmodern systemic perspective, however, the therapist is also a node in a network of relationships that influences how he or she gives meaning to what he or she perceives. His or her thinking, just like that of his or her clients, is socially constructed and therefore not more (or less) “true” than that of the client. The idea of the psychotherapist as an expert in the client’s life is abandoned and there is a shift toward psychotherapy as co-construction (Gergen and Warhus, 2001). In this, the therapist’s task is two-sided. On the one hand, he or she is responsible for creating a context in which clients feel safe enough to share their stories. On the other hand, from a position of not-knowing (a position characterized by curiosity and reflexivity, whereby the therapist is open to learning about the client and does not assume he or she “knows” what the client’s problem is too quickly), it is his or her task to facilitate a dialog in which new, more viable meanings can be generated (Rober, 2005; Anderson, 2012). Thus, the focus of the systemic therapist is not on “discovering” or “uncovering” experiences as such, but on the effect, the therapeutic dialog has on the client both inside and outside of the therapeutic room.

Although embodiment and non-verbal interactions have always taken central stage in systemic therapeutic practice (Jackson, 1957; Satir et al., 1991; Wilson et al., 2020),^2^ recent theoretic developments with their focus on language and stories seem to have created a “gap” between systemic theory and its practice (Bertrando and Gilli, 2008).^3^ We propose that this is where the EEC framework can be of help.

The Embodied and Enactive account of Cognition (EEC), two pillars of the “4E Cognitive Science”—see Newen et al. (2018), is an account that has a number of core concepts very much in line with systemic thinking, and so, could enrich systemic theory. EEC opposes classical cognitivist and computational views of cognition that reduce cognitive processes to manipulations of information in the brain. Cognition, it is argued, is grounded in broader perceptual and sensorimotor systems and develops in interaction with the environment. “Embodied” cognition refers to the idea that cognition is not limited to what happens in the brain but includes processes both in and outside of the brain. “Enactive” cognition refers to the idea that cognition involves more than just the body; it involves explicit, adaptive, and reciprocal interactions between the agent and its environment. Often EEC proposals include the notion of affordances from ecological psychology. Affordances are possibilities for action offered to an agent by the environment that emerge in their relation.

Perhaps unsurprisingly, body-oriented and art therapies have quickly found their way to this new approach to cognition as a foundation and inspiration for their practice (Röhricht et al., 2014; Samaritter and Payne, 2016). For therapeutic approaches that emphasize movement and non-verbal interaction, such an approach to cognition offers a ground on which to base the therapist’s actions and clarify how therapeutic change takes place. But what about systemic collaborative therapy with its focus on language and dialog?

Here, we point out that EEC can extend to linguistic practices as well. Language, according to enactivists, is a stream of activity in a sociomaterial world of practices (Di Paolo et al., 2018). It is not a disembodied or decoupled activity of higher minds, but one that emerges in social interaction. Enactivists refer to the term languaging to talk about activities connected to language, including speaking, but also non-verbal behaviors like gesturing and mimicry. Languaging captures the “continuity between bodily engagements and activities including speaking and verbal behaviors” (Jensen, 2014, p. 6). Language is therefore a cultural process, grounded in human biology and sociomaterial practices that captures both the emotional and the affective dimension of human interactions. This allows systemic collaborative therapy to draw from EEC, as it finds support there to the idea that dialog is an embodied activity as well.

We will now briefly look at the role of metaphors in classical and systemic psychotherapies, followed by an analysis of what we can learn about metaphors from the EEC perspective.

## Metaphors in Psychotherapy and Systemic Collaborative Practices

In the psychotherapeutic literature, the term “metaphor” is usually given a very broad meaning. Metaphor simply refers to “a way of speaking in which one thing is expressed in terms of another” (Kopp, 1971, p. 28). Reflection on the role of metaphors in psychotherapy has a long and rich tradition (see, e.g., Tay, 2013). In an attempt to structure the multitude of ways in which metaphors can influence therapeutic processes, Lyddon et al. (2001) proposed the following classification: (1) relationship building between therapist and client, (2) accessing and symbolizing client’s emotion, (3) uncovering and challenging client’s tacit assumptions, (4) working with client’s resistance, and (5) introducing new frames of reference.

According to Tay (2016), the literature on the use and role of metaphors in therapeutic processes shows two main trends: a therapist-centered approach and a client-centered approach. In a therapist-centered approach, the focus is on the therapist as the author of the metaphor. It examines and describes what the therapist needs to be attentive to in devising metaphors, bringing metaphors into dialog with their clients, and using metaphors in therapeutic processes. Some therapeutic movements see in the layering of metaphors an opportunity for therapists to communicate indirectly to the client’s subconscious and thus avoiding conscious resistance (Erickson and Rossi, 1976; Roffman, 2008; Burns, 2012). However, the literature also describes ways in which metaphors are used more explicitly. Metaphors offer therapists a way of discussing often abstract hypotheses, ideas, or advice with clients in a concrete manner (Lakoff and Johnson, 1980; Tay, 2013, 2017). In an attempt to support therapists in this, there are manuals that provide numerous metaphors that can be used by therapists when they deem it useful or necessary (Blenkiron, 2011; Stoddard and Afari, 2014).

The literature from a client-centered approach to metaphors focuses primarily on the client as the author of the metaphor. This approach is in line with more non-directive approaches within psychotherapy and sees metaphors as means for clients to verbalize what they think, feel, and experience (Lakoff and Johnson, 1980; McMullen, 1996). The focus is mainly on the content of the metaphor, as the therapist interprets the client’s metaphor from his or her own frame of reference. The metaphor is thus seen as a gateway to uncover psychopathology (Rhodes and Jakes, 2004; Coll-Florit et al., 2021) or unconscious thoughts and feelings (Borbely, 2008). However, a more process-oriented approach, like systemic collaborative therapy, emphasizes the importance of bracketing one’s own interpretations and adhering as much as possible to the client’s process and meaning making (Kopp and Craw, 1998; Sims, 2003; Rucińska et al., 2021). From the systemic collaborative therapeutic perspective, metaphors can be generated by both the client and the therapist (Rober, 1999). Regardless of who generates the metaphor, the metaphor serves to co-construct new stories. The metaphor in itself does not conceal any “truth,” nor is it intended to convey “knowledge” or “insights,” but can offer an approach to investigate experiences from a different perspective.

Although metaphors are an integral part of psychotherapeutic practice and a great deal of research has been conducted and written about the role of metaphors in psychotherapeutic processes over the years, Tay (2017) is concerned about how little attention is paid to how contemporary metaphor theory can enrich psychotherapeutic practice. This inspired us to ask: what is it about metaphors that makes them good therapeutic tools? Our answer to this question will be situated within the EEC approaches to metaphor—particularly the enactivist approach, which sees metaphors as tools for joint meaning-making practices. To understand the processes of meaning-making in metaphoric engagements, we now turn to EEC, which sees metaphors as embodied, enacted, or ecological, and discuss how it can enrich and underpin the use of client-generated metaphors in systemic collaborative therapy.^4^

## Embodied, Enactive, and Ecological Metaphors

In the philosophical literature, metaphor is typically seen as a literary device that involves conscious violation of established categories, allowing us to understand one kind of thing (often abstract or unfamiliar) in terms of another (more concrete and familiar; Lakoff and Johnson, 1980; Stilwell et al., 2021). Recently, however, metaphors are no longer treated as linguistic entities (figures of speech) or linguistic processes (source-target mappings) alone but are seen as cognitive and imaginative processes (Gibbs, 2006), perceptual processes (Szokolszky, 2019), or even as affordances and figures of action (Jensen and Greve, 2019). For instance, the dynamic view of metaphor (Müller and Tag, 2010) proposes a dynamic intertwining of social, cognitive, and affective processes in metaphor production and understanding, and systematically integrates social and cognitive processes for the analysis of metaphor activation in conversational interaction. In this paper, we do not aim to provide a comprehensive overview of all of the available proposals on how to think of metaphors. We will, however, provide a brief characterization of some of the most recent embodied, enactive, and ecological (Machielsen, 2019; Szokolszky, 2019; Stilwell et al., 2021) approaches to metaphor, so as to situate our own, dialogical-enactive approach to metaphor use in therapy.^5^

We will start with the claim that metaphors are embodied—an idea so popular that it should not be controversial. But what exactly does it mean for the metaphor to be embodied? The literature on metaphors refers to different senses of embodiment, which is worthwhile mapping out. By saying that “metaphors are embodied,” one could, in the least, mean one of the following things: metaphors have sensorimotor roots, rely on motoric simulations, require activation of physical circuits in the motor cortex, or involve affective bodily processes—and this list is not exhaustive. We will try to unpack some of these different meanings at play.

For instance, the concept of “embodied metaphor” can refer to the fact that existing metaphors have bodily roots, as seen in the examples of Lakoff (2008, 2012) and Lakoff and Johnson (1999). They propose that while metaphors are linguistic tokens, the language that people have developed is grounded in their physical experiences, and conceptual metaphors can build on embodied experiences. According to Lakoff (2008), all complex conceptual metaphors can be decomposed into primary metaphors, and primary metaphors are acquired “by going about the world constantly moving and perceiving” (Lakoff and Johnson, 1999, p. 57). For instance, “AFFECTION is WARMTH” is a primary metaphor, whereby we associate physical warmth with friendliness. This is so, according to Lakoff, “(b)ecause primary metaphors are persistent (long-lasting or permanent) physical circuits in the brain” (Lakoff, 2012, p. 782, see also Kompa, 2017, p. 203).

However, sometimes our body not only serves as a grounding for metaphoric thought, but also seems to serve as reference for understanding creative and unique language use. Metaphors trigger bodily responses which enable us to “experience” the meaning of a metaphor. For instance, according to Raymond Gibbs (2006), to understand metaphors containing an action verb is to actively imagine oneself engaging in that very action. And according to Littlemore (2019), embodied metaphor is characterized by a visceral bodily experience used to describe something abstract. Embodied metaphors can have a strong physical association or elicit strong physical reactions. Here, “embodied” is used to describe situations when one has an overt, physical response to words.

According to Littlemore (2019), metaphors differ in the degree to which they are embodied. There are metaphors that elicit a neurological response, there are metaphors that can appeal to our experiences with, and our knowledge about, our bodies, and there are metaphors that can also be understood because they are part of our language or convention, as they grew from the history of our embodied experiences and interactions. The sense of embodiment that we wish to emphasize, however, is that there is an explicit role for the body in shaping metaphor understanding, connected to enaction: occurrent bodily movement and activity. We will now turn to the enactive proposal.

We take Gallagher and Lindgren (2015) as providing a good example of an enactivist take to metaphors. As they write,

The term enactive here signifies not a different kind of metaphor per se but a different kind of engagement with metaphor. Specifically, we can say that an enactive metaphor is one that we enact—that is, one that we put into action or one that we bring into existence through our action (p. 392).

They contrast enactive metaphors to sitting metaphors (ones found when reading a book, which are “sitting” on the page and waiting for the reader to discover them). Instead, enactive metaphors “do things, but only when we engage with them in some fashion” (*ibid.*, p. 392). Their focus is on how we can put metaphors to work in actual learning situations and provide an example of enacting metaphors in specific contexts.^6^ Enactivist proposal, in short, is that metaphors are achieved in participatory action, their meanings are created in the interaction, and they are a part of joint construction of a new reality (Rucińska and Reijmers, 2015).

From the ecological approach, Jensen and Greve (2019) propose a somewhat similar account of metaphors as a products of organism-environment systems. They see metaphoric engagement as “a form of doing that is embedded in the ways that we do things in the world, and as such it can be understood as skillful manipulations of environments of any kind” (p. 2). They propose to thinks of metaphors through the concept of affordances, which has the advantage of helping us “to focus on metaphor as part of our active doings, rather than as inner mental processes or stylistic features” (p. 12–13). They further speak of levels of metaphoricity of words, to contrast the idea that literal and figurative meanings are sharply distinguishable. They propose that

metaphoricity needs to be seen as a scalar value; something that is more or less activated or present (…). Notably, metaphoricity is not restricted to (the meaning of) words or verbal actions but [relies] on a variety of bodily activities and sensations (2019, p. 10).

Both enactivist and ecological proposals notice that the metaphoric meanings come about in interactions. Enactivists argue that we construe our meanings through participatory sense-making, an idea that meaning is jointly achieved through a history of breakdowns and recoveries in interactive coordination (De Jaegher and Di Paolo, 2007). We actively, in an ongoing fashion, make sense of our environments: we engage in interactive processes of bringing forth or enacting of a world of relevance. Ecological take to metaphors as affordances allows metaphors to be part of such meaning-making practices.

The embodied-enactive-ecological proposals underlie the view on metaphors that we share. Where our proposal differs from the rest is in thinking about how it is that enacting metaphors actually brings about change. We now turn to an analysis of how EEC metaphors are supposed to work, followed by our proposal of enacting metaphors in therapeutic dialog.

## How Do Metaphors Work?

We have spelled out above what the gist of the embodied, enactive, and ecological take to metaphors is. But that is not yet sufficient to explain how metaphors work, or what it is about being embodied, enacted, or ecological that allows metaphors to provide us with new meanings. In this section, we attempt to provide a short overview of how metaphors are supposed to work on the embodied, enactive, and ecological models, each analysis followed by a critique or worry pertaining to that method. We then propose to fill a gap in the available explanations with our own proposal.

One way in which metaphors are said to work is because they allow us to see similarities between two entities that are normally considered distinct (Szokolszky, 2006, 2019). This is a skill that, from a representational lens, is a complicated cognitive task (Norbury, 2005).^7^ But it can be well accounted for by ecological psychology. Ecological psychologists speak of direct pick-up of an invariant pattern in an informational array specific to both the source of the metaphor and its target (for example, the informational array specific to both fireworks and flowers that allows one to metaphorically consider fireworks as “flowers on the sky”).^8^ According to Agnes Szokolszky (2006), prototypical metaphor is based on a clearly perceivable shared resemblance (and/or “shared affordances”) between two objects. Finding a resemblance lies the ground for a metaphor as it creates a possibility to perceive one thing in terms of another (e.g., a shoe in terms of the boat), and in turn, to say something metaphorically. This view roots the capacity to act on metaphors in simple perceptual, not complex cognitive processes, such as counterfactual thinking or being able to represent one thing as another. So while metaphor was believed to presuppose classification skills not available to the preschool child, the ecological perspective considers metaphors as learning tools that even young children can develop, because from the ecological perspective, metaphoricity is a perceptual phenomenon.

While stepping away from over-intellectualizing the mechanisms underlying metaphoric engagements is something we share with the ecological approach, we think that metaphor sources and targets do not need to share structural similarities, and resemble one another in any way. Many metaphors are conceptual (e.g., “love is a battlefield”) and do not allow for any perceptual informational patterns to be picked up on. And sharing of affordances is much too broad of a claim, as metaphor sources and targets can share an infinite number of affordances (possibilities for action), such as possibility for discourse. This is not something that makes the perceptual view special in explaining how metaphors work, at least, not in systemic collaborative therapeutic context. Also, while ecological psychologists can say that they only speak of prototypical, not conceptual metaphors, there is another issue at hand: passivity of this approach. In therapy, the stress is on the active role of dynamic action and interaction in relation to affordances for metaphoric discourse. The act of simply seeing resemblances between the metaphor and its source, or being mindful of the bodily origins of metaphors, is not enough. In our proposal, we will focus on acting on metaphors. In this respect, we do not see metaphoricity as requiring perception of resemblances or perception of “shared affordances,” but we propose to think of metaphors as tools for new ways of seeing and interacting.

Another way embodied metaphors are said to work is because they refer to our personal, lived experiences (e.g., Littlemore, 2019). These metaphors are based on experienced correlations between the source and target domains, and not on perceived similarities. Experienced correlations refer to our past embodied lived experiences and feelings, thereby being more likely to neurologically trigger us when we hear them.

One concern with the claim that metaphors are grounded in bodily experiences is that it seems to suggest that these metaphors would trigger personalized, individual understandings, as each metaphor is based on one’s own, individual, embodied experience. This would face the worry that the metaphors would not be understandable to others. However, this view is not a problem if combined with enactivism and participatory sense-making. Participatory sense-making is partially grounded in our bodily experiences, but mainly, it suggests that meaning-making is a joint act, one that develops intersubjectively. Our social interactions change how we perceive and understand the world. No individual person chooses their own meanings of experiences; experiences become meaningful in interaction with the others, negotiated within a cultural practice. What this means in practice is that the meaning of a metaphor is flexible and takes shape within the context in which it is used. One and the same metaphor could even be “reused,” as we will show below. We build on this insight to discuss how metaphors get their meanings in the context of a therapeutic dialog in the next section.

Yet another way embodied metaphors are said to work is because they trigger sensorimotor responses, as captured by some of the embodied proposals (Schaefer et al., 2015; see also Kompa, 2017). Here, the source and the target of the metaphor need not be alike in any structural way; what happens is an activation of the brain regions that would also be activated should we actually experience what is expressed in the metaphor. Kompa (2017) happens to call this “simulation,” even though he refers to sensory-motor processes involved in the re-enactment of an embodied experience.^9^ Consider explanation of Kompa (2017) of how metaphors work by simulation:

So the idea, basically, is that in order to understand a linguistic expression one has to simulate the corresponding experience. When I hear the word “grasp” I simulate (reenact) the action of grasping. And since I have grasped before, I will be successful and come to understand the word in question. Simulation, in turn, requires activation in sensory and motor (as well as affective) regions of the brain because in simulating a particular experience we exhibit roughly the same pattern of neural activity that accompanied the initial experience. Language comprehension crucially involves recruitment of the sensory-motor system (p. 196–197).

We agree with the claim that language comprehension is a sensory-motor endeavor. There is a multitude of evidence from neuro- and cognitive science to support this, including the fact that some action verbs elicit activation of motor cortex in the brain (Barsalou, 1999; Hauk et al., 2004).^10^ However, Kompa (2017) argues that simulation (as described above) fails in accounting for metaphor understanding because it “provides us with a theory of literal interpretation. But interpreting metaphors requires that we leave the literal meaning behind (…). Understanding metaphors requires that one looks at things differently than one did before” (p. 206). Consider the metaphor “to GRASP an IDEA.” Is activation of the same neurons when one actually grasps a cup that which allows us to understand the metaphor in question? Kompa challenges this, arguing that simulating (or in EEC terms, *reenacting*) our previous bodily experiences, by means of activating the related sensorimotor processes of grasping when thinking about the metaphor “to GRASP an IDEA,” does not yet get us to understand its metaphoricity.

While Kompa’s challenge should be analyzed in more detail in the future, we suggest that for therapeutic purposes, the idea of mere activation of sensorimotor circuits connected to action words during speaking of those words is indeed not central, as it is not in the triggering of sensorimotor processes (such as the sensorimotor activation of hand-grasping) that the enacting of the metaphor takes place—it is in the dialog. If we were to work with the metaphor “to GRASP an IDEA” in systemic therapy, it would work as a metaphor because grasping activity is connected to the wider network of meanings we can associate and create with the action word “to grasp,” such as a feeling of achievement or the emotion of success. In our view, it is what one can do with the metaphor to bring about change that makes the metaphor work, as we will explain below.

Thus, while there are several pathways through which metaphors can work, each way tailored to its own context,^11^ we will focus on idea of Gallagher and Lindgren (2015) that metaphors work because they are enacted: they invite us to “act out their understandings with [our] bodies” (p. 398), instead of thinking through the mappings from source to target domains. As Gallagher and Lindgren note, enactive metaphors are not a different type of metaphors—the difference lies in how we use them. Enactivism can thereby propose a different way of looking at how metaphors can be put to action, in dialog, which we think is most productive for a therapeutic context.

We endorse this enactivist approach to enacting metaphors and are sympathetic to the abovementioned enactivist proposals. However, one limitation of the available proposals is that they treat enaction rather literally. Gallagher and Lindgren (2015) speak of enacting metaphors in acts that are embodied performances of pretense: it is in the moving of one’s body that the actors are acting metaphorically. Jensen and Greve (2019) say that enacting happens “in-and-through the gestural movements” (p. 18).^12^ And most recently, Stilwell et al. (2021) discuss enacting metaphors of pain in therapy and propose that the metaphor is enacted when manual pressure is applied by the therapist to the patient’s pain source. Not before, not after, but in the touching is when the metaphors were enacted.^13^ In all of these examples, the metaphor is literally performed, with movement, gesture, or physical touching. Stilwell et al. even explicitly contrast enactive metaphors to verbal metaphors; the latter are seen as part of “passive patient education” (p. 243). This is a rather limited view of enacting, as enactivism extends to language as well. So while we agree that sometimes this is how we can enact metaphors (just as we can explicitly enact our imaginings in performances of pretend play—see Rucińska and Gallagher, 2021), our point in this paper, and the adjustment to the available enactive views of metaphor, is that enacting of metaphors can also happen in the talking, as talking is also a kind of a doing. We propose that no explicit performance aside linguistic verbalization needs to take place for the metaphor to still be enacted, for language is also an embodied and enactive process. In the next section, we clarify how we think enacting metaphors works in dialog.

## Enacting Metaphors in Dialog: Examples From the Therapeutic Practice

Following the analysis above, we propose that in systemic therapy, we can enact metaphors in dialog. What this means is that, in a therapeutic context, a therapist can use action words and verbs associated to the metaphor to bring about new action possibilities for the client who introduces the metaphor. The therapist does not need to analyze the meaning of the metaphor; he or she creates new meanings with the metaphor as introduced by the client to make together new sense of the client’s situation.

Take, for instance, talking about depression. Such diagnosis, on its own, does not warrant new behaviors. The narrative of “depression” is static and often restrictive: once diagnosed with it, it can have a debilitating effect on a person (one does not know what to do about it but accept its consequences). But when a client seeks therapy in order to cope with his/her depression, it could be helpful for the client and the therapist to speak with metaphors. This could work, because action-oriented verbs can be easily associated with such metaphors. The therapist can skillfully engage and play with metaphors (more than with clinical diagnostic words) to bring about new conversations with the client. We will show this in the example below.

Our point for now is that metaphors allow for a transformative experience when they are enacted in dialog. But that enaction needs not be a bodily performance. Conceived of as linguistic affordances, or possibilities for future actions enabled by language, metaphors are dependent on use. Metaphors in therapy can function as objects or tools for manipulation. So while metaphors could work due to their symbolic properties, we want to highlight another way metaphors work that has not been discussed in detail before, which is that they work when they act as linguistic affordances for action. To act as linguistic affordances for action means that they open up new ways of languaging, talking about, and making sense of things.^14^ Below, we will provide two examples from the systemic therapeutic encounters to illustrate our point.

### Case One: Andy

Andy is 15 years old and has been struggling with a morbid form of obesity for several years. His parents divorced when he was 10 years old. He remembers how, shortly after the divorce, he started eating more and more. Eating, he says, offered a form of comfort. His mother was on her own after the divorce. In order to cope financially with the new situation, she worked long hours. Therefore, Andy was often home alone. Initially, Andy was not really bothered by the weight he was progressively gaining. However, this changed with the transition to secondary school. There, he was bullied and excluded because of his weight. Young people hurled words at him such as “elephant” and “fatty.” He became increasingly ashamed of his appearance and locked himself up in his room. He hid behind the computer and joined an online community of gamers. His unhealthy eating habits and sedentary lifestyle led him to develop morbid obesity. Any hope of change faded away like snow in the sun. He felt trapped in his own body and powerless over his situation. It became increasingly difficult for him to go to school and endure the looks of other young people. When Andy was 14 years old, his mother suggested he seek help from a medical pediatric rehabilitation center where obese youngsters receive multidisciplinary residential treatment to help them re-establish healthy eating and living habits and regain their self-confidence. It took a year before Andy dared to take the step.

During one of his conversations with Andy (A), his therapist (T) explores the way in which the looks of others have affected him.

*T: I remember from one of our previous conversations that you were often excluded and bullied at school. Despite that difficult situation, you continued to go to school. How did you do that?*

*A: Over time, I [put on] a harness. That harness helped me cope with all those words. It was the only thing I could do against all those ugly things people said to me.*

*T: So you put a harness on as a way of protecting yourself? Is that right?*

Notice how Andy spontaneously uses a metaphor to respond to the therapist’s question. The metaphor is not chosen randomly. Andy is a gamer and plays games in which a harness is often used to protect your avatar against a dangerous virtual world. His experiences with gaming offer him an arsenal of images from which he can pick and choose in order to express his experiences in the conversation with the therapist.

The therapist wants to further explore, from the not-knowing position, Andy’s experiences of going to school. He decides to do so by “staying with” the metaphor that Andy has given him (by continuing to use and to explore it) and asks Andy further questions:

*T: When you are wearing your harness, do you no longer feel the [weight of the hurtful] words?*

*A: Yes, but just a bit. If you are hit [by those words], you still feel the blow. [With the harness] it just feels less painful.*

*T: OK, so you still feel the blow of the words, but it is muffled.*

*A: Yes, indeed.*

*T: And what was it like for you to have to walk around with such harness?*

*A: I locked myself up in my room as much as possible so that I didn't have to see anyone. That way I didn't have to put on my harness.*

*T: And why was that exactly? What was it like for you to have to walk around with such a harness on?*

*A: It was very tiring to have to keep it on all the time. It took a lot of energy not to let people see how I felt inside. Even though it varied from day to day.*

*T: Oh yes, and what did that [variety] have to do with?*

*A: Partly [it was] because of the number of beatings I had to endure. And at the end of a school week, the harness also felt much heavier than at the beginning of the week.*

From a therapeutic perspective, we could say that Andy uses the image of a “harness” as a metaphor for his struggle to shield himself from painful experiences. He uses the metaphor as a mean of expressing how he coped with the bullying. The concrete image of the harness helps him put into words the complexity of his experiences. However, from an enactive perspective, the metaphor provides a “shared space of affordances” (Gallagher, 2020, p. 113), within which Andy and his therapist can move to further explore his experiences. Because a harness is something tangible and concrete, both Andy and the therapist can refer to the multisensory aspects connected to using or wearing a harness to illuminate Andy’s experiences. Harnesses can be taken off and put on; they can be heavy or light (this can vary from day to day or moment to moment); and they can absorb the blows from outside, though some blows may still feel harder than others.

Through his questions about the harness (how it feels, or what actions it allows), the therapist invites Andy to go beyond a static comparison. Andy’s experiences of bullying are contextualized and differentiated within the language of the metaphor. In and through this metaphoric dialog, a noticeable shift in meaning takes place. In contextualizing and differentiating, Andy increasingly appears to the therapist as a young man who has worked hard to hold his own in a “battlefield” of remarks and hurtful words. He is not just a “harnessed” young man, hiding behind a shield. He is a young man who puts on “an armor,” takes blows and braves fatigue. Where the metaphor initially afforded hiding, now it also affords resisting and fighting back. By shifting the focus from a static comparison to a dynamic exploration, the therapist laid the building blocks for Andy to increasingly see and experience himself as a young man who acts, interacts, and makes choices—a young man with agency. This is also tangible in the following excerpt:

*T: I hear you talk about how you had to take blows and how tiring it was to wear a harness. And yet it took you a year to take the step to our center. Could you help me understand that?*

*A: I took the step only after it became too tiring to wear the armor.*

*T: And why not before?*

*A: I thought I could keep the armor on. When I felt that was no longer feasible, I took the step to the center.*

*T: So you only took the step to our center when you felt you could no longer keep it on. What perseverance!*

*A: Maybe (laughs).*

In working with metaphors from a systemic collaborative perspective, the therapist is careful not to analyze the metaphor but rather to create a space in which clients can share and explore their experiences in such a way that new facets of their experiences can be approached and illuminated from a different perspective. In the excerpt, the therapist highlights Andy’s perseverance in carrying the weight of the harness, and thus invites Andy to reconsider his experiences from a different perspective. Andy seems to accept the proposal and creates a space to generate new stories about himself together with his therapist.

After an exploration of what helped Andy to show that perseverance, in the next excerpt, the therapist invites Andy to explore what has changed since the start of the obesity rehabilitation program. Notice how the therapist again poses his starting question in such a way that it invites Andy to talk about himself as someone with agency, someone who makes choices and takes well-considered actions.

*T: At some point you took that step [of coming to the therapy]. And then? What did you do with the harness?*

*A: I kept the harness on for the first few months. When I got to know the group better, I took it off so they could see more of who I really am.*

*T: Did your experiences with the group give you the courage to take off the harness?*

*A: Yes.*

*T: And what did you notice that made you confident that you could take off the harness?*

*A: I felt at home in the group. I felt that I did not have to focus on how I came across and that I could just do as I did at home. I felt that I did not have to pretend to be someone I am not.*

*T: And could you tell me more about taking off the armor? How did it go?*

*A: It was piece by piece.*

*T: Piece by piece? And what helped you to take off the next piece after each one?*

*A: I just noticed that nothing much changed. Everyone kept looking at me in the same way and continued to deal with me in the same way.*

*T: And that gave you the confidence to take off your harness piece by piece?*

*A: Yes*

*T: And now, where is your harness now?*

*A: It is hanging on the coat rack. Gathering dust. You never know when I might need it again.*

*T: (Laughs). So you haven't thrown it away yet?*

*A: No.*

Initially, the therapist created a conversational space in which Andy could talk about his experiences in taking the blows and carrying the weight of the harness. The focus was on Andy’s burden. As the conversation progressed, the therapist shifted to a more action-oriented exploration. He invites Andy to talk about what Andy has “done” with the harness and why. The focus of the therapist is now on what Andy has experienced and the way he has responded to it as an active participant in the event. The therapist’s questions invite Andy to explore the way he handled his situation as somebody who critically evaluates and makes informed choices. He chose “putting on a harness” in order to defend himself, he chose to wait with an admission to the rehabilitation program, and when he could no longer continue by himself, he chose to “leave the harness on” in the first few months, after which he decided to gradually “take the harness off,” piece by piece. The action-oriented questions invited Andy to no longer see himself as someone who just suffered, but also as someone who handled the situation the best way he could.

Although the therapist deliberately chose his questions and “scaffolded the conversation” (White, 2007), he did not unilaterally determine which direction the conversation would take and which meanings could be drawn to the foreground. Within the context of the therapy, the conversation was a process of participatory sense-making in which both participants influenced how the conversation proceeded. By means of an enactive metaphor, Andy and the therapist co-created new storylines in which Andy appears as an active participant in the various nodes of relationships in which he is entangled. Inspired by this newly found relational sense of agency, it hopefully encourages him to re-position himself differently in the various nodes of relationships in which he is entangled: not just as a victim of bullying, but also as a courageous and powerful person.

### Case Two: Ben

Ben is a 17-year-old young man with autism. He struggles with negative thoughts. The struggle does not always show on the outside—it takes place “in his head.” The negative thoughts do not let go of him. During one of the conversations, Ben tells the therapist about his self-injurious behavior: sometimes, when he is having a really hard time, Ben cuts himself. He tells the therapist how, when he is having such a hard time, he feels both the need to be left alone and he wants to be helped. Just before he cuts himself, the feeling of being left alone dominates. After he has cut himself, he realizes that he needs help. Only, he does not know how people could help him.

The therapist is curious about this shift in experience and asks Ben what happens after he cuts himself that he feels the need to be helped. Ben tells the therapist how cutting helps him to let his thoughts come out. When his head is overflowing, he cuts himself. And at that moment, he realizes he needs help. The therapist tells Ben how striking he finds the fact that the cutting brings about a shift in his experiences, from a need to be left alone, to a need to be helped. Ben acknowledges this, but initially finds it difficult to put into words what exactly makes that shift happen. After a short silence, Ben suddenly says as:

*B: It is like with tattoos. If you are against tattoos, you find them a form of self-mutilation. But if you like tattoos, a tattoo can be seen as a story that you carve into your body. The cutting works for me like a tattoo works for [them].*

*T: I hear you use the word "story." A tattoo is a story that is told. (…) And what is the story behind your tattoos?*

*B: One big story of the last two years.*

*T: And does it have a title?*

*B: I do not know if I could give it a title.*

*(…)*

*T: Can I ask you a strange question? Let’s say we would meet again in a year and you put a tattoo over your scars? What kind of tattoo would you choose? What form would it have?*

*B: That is a difficult question. I think I would put an image of a skeleton in a dinner jacket dancing a slow dance with an ordinary lady. In fact, that might also be the title of my story.*

*T: The skeleton in a dinner jacket slow dancing with a lady?*

*B: Yes.*

*T: Ok, and what is the story behind that tattoo?*

*B: The lady could be anyone who self-mutilates for one reason or another. And she slow-dances with death. Because you never know when you cut too deep.*

*T: Why did you choose a lady?*

*B: Because I see myself as the skeleton rather than the lady. At times I feel very close to death.*

*T: It says something about how close you are or were to death?*

*B: That's why it is a slow dance, because at times I feel so close.*

*(…)*

*T: So in a year’s time that tattoo would be a story of how close you were to death, and how intimate that dance was at the time. And perhaps how brave you were too?*

*B: Yes, probably.*

While discussing the metaphor of the tattoo, the therapist made some choices in what questions he asked and which ones he left out. The questions he asked were informed by his therapeutic hypotheses as well as by Ben’s answers. Working with the “tattoo” metaphor initiated by Ben, the therapist could have continued the discussion in many action-oriented ways. For instance, the metaphor could have been used to posit different kinds of questions pertaining to agency, such as: “Is the tattoo a finished product?,” “What colors will it have in a year?,” “How visible could you make it for the outside world?,” or “Will you cover it up or leave it visible?” Such questions open up the space for Ben to think about his future. Even though “tattoo” is a noun, and it is most easily attributable to Ben’s scars, the therapist has the tools to push the meaning of the metaphor further, to induce in Ben thoughts about taking control and acting in the future. Working with the metaphor of a tattoo gives the therapist the room to explore possibilities for action that are not available, when one receives, in similar circumstance, a diagnosis such as, e.g., “Ben is depressed.”

How do these two examples relate to enactivist idea of sense-making? In Andy’s case, talking about the “harness” helped him put into words the complexity of his experience and allowed a new sort of conversation to unfold, one that helped Andy make new sense of his hurtful experiences together with the therapist. Similarly, in Ben’s case, talking about the “tattoo” allowed him to feel understood, as it allowed for the conversation to go forward with the therapist, where they made new sense of his situation together. The value of both metaphors is that they paved the way toward different conversations and in turn, allowed gaining new perspectives on one’s problems—perspectives that, thanks to action-oriented discussions that followed, allowed both Andy and Ben to feel in control over their problems. We will elaborate on these added values of enacting metaphors below.

## What Makes Metaphors Good Therapeutic Tools? An Analysis

From our dialogical-enactive perspective, metaphors are good therapeutic tools not because they uncover experiences, but because they allow sharing and changing those experiences. Talking of “uncovering experiences” is problematic in our view, because it suggests that there are hidden truths that can be uncovered with the metaphor, truths that were somehow “repressed” and are now brought to the surface. So while it is possible that one gains new insight from enacting metaphors, this is not the goal of systemic collaborative therapy, as the new insight should be seen as yet another perspective the client can take on their problem. We see metaphors as tools that afford the therapist different possibilities for making the client feel understood. They are also good for exploring client experiences and bringing new ideas to the foreground. Metaphors affords telling new, different stories, and in the questions a therapist asks, he/she can bring forth in the client experiences of agency. Finally, metaphors function as lenses through which the client, after the therapy, can approach and experience the outside world.

How do the metaphors do that on our picture? In short, metaphors work when they are skillfully oriented toward actions in dialog. The metaphors that Andy and Ben shared were oriented toward action by the therapist. And even though their metaphors were nouns, the systemic-enactive approach utilized by the therapist made him focus on possible actions associated with these nouns. Harness is something one can use to hide behind or to fight with. Tattoo is something one can color, draw, or with which one can tell a story. From the systemic-enactive perspective, any metaphor (even “sitting” metaphors) can be traced to an action. The content of the original metaphor is not as important as what one does with the metaphor. The best way to bring about change is through action words, through which the therapist can bring about change when he invites the client to collaboratively do something with the metaphor.

Why does enacting metaphors with action words work in psychotherapy? To repeat, it affords the client and the therapist ways to explore experiences in such a way that the clients can feel heard and understood, communicate how they think or feel, and be invited to explore their agency—and not just help the client provide an insight on their condition. While clarity is important, from the systemic perspective, insight is not enough for progress (as Watzlawick, 2009 famously said, “insight may cause blindness”). Also, the choice of the metaphor, by itself, does not lead to a transformative experience. Making an analogy to capture one’s experiences in itself is not sufficient, for it is not the metaphor itself (thanks to its symbolic or embodied properties) that does the work, but how the metaphor is used and responded to (or enacted) in an interaction. On our view, it is the communicative act with the metaphor that becomes a transformative experience for the clients. The metaphor has to be received by another, accepted, and acted upon in return. Metaphors work best when they are part of collaborative engagement.

Take the example of Ben, who has used the tattoo metaphor to talk about his struggles. We noticed that when Ben used the same metaphor to talk to his mother, he felt not understood. From our perspective that is because without his mother picking up on the metaphor in their conversations or in any way acting on that metaphor, the communication between them stops, and the engagement does not result in a transformative experience for Ben. However, the metaphor does work with the therapist, who uses it to further the discussion. To the systemic therapist, metaphor is a vehicle for change.

In short, metaphors themselves do not do the heavy lifting. It is important what one does with the metaphor, not what the metaphor means. It is how the therapist enacts metaphors by discovering action words that allows the client to move beyond the metaphor that makes the metaphor therapeutically useful. Consider for contrast the Big Book of ATC metaphors (Stoddard and Afari, 2014) that proposes to provide an “exhaustive list of metaphors” geared toward treating various conditions, ranging from anxiety, depression, trauma, or an eating disorder. We oppose the idea that finding the “right” metaphor will help one solve his/her problem.

Finally, focusing on action words allows the client to regain a sense of agency. When metaphors are discussed with action words, a therapist can choose to elaborate the metaphor with questions that a client, in answering them, can regain a sense of influence over his or her life. We see sense of agency as more than just knowledge over the kind of influence one has; it is an embodied experience of being able to act, to do, to move, and to change. Metaphoric engagements can offer the possibility to explore one’s experiences in a way that one can feel that change is possible. As the therapist wants to give his/her clients the feeling of having a grip on what they are struggling with outside of the therapeutic room, the hope is that the sense of agency will stay with the client after the therapy ends and translate into coping in everyday life.

## Conclusion and Follow-Up

To summarize, we have presented in this paper a proposal for seeing metaphors as tools for action in systemic collaborative therapeutic context. We have argued for the view that metaphors work best when they are enacted, and that engaging in explicit movements or performances is not the only ways to enact metaphors. Metaphors can also be enacted and jointly explored in dialog. As the intention of the systemic collaborative therapist is to bring new experiences for the client, metaphors are useful resources for systemic collaborative therapists, when they are enacted with action words. Enacting metaphors allows bringing forth new perspectives and a sense of agency in their clients, because the therapist dynamically co-constructs the metaphor with the client and uses action words to jointly create new meanings. Thus, our answer to the question “what makes metaphors good therapeutic tools?” is that they can be connected to action words, through which the client’s embodiment and agency can be explored.

While our analysis is based on the collaborative systemic approach to psychotherapy, we are convinced that other therapeutic models can also be inspired by it. An important condition, however, is that the focus of the therapy is not on discovering or exposing “fixed,” “hidden,” or “repressed” meanings, but on creatively co-creating more liveable realities, which is why not all talking therapies may benefit from our intervention strategy.^15^

We will share three practical insights that follow from our proposal. First, on our view, one does not need to work with a culturally established metaphor: any word will do. The therapist should keep an open eye for words that afford enactive metaphoric explorations. For example, it is by using regular words (such as “tattoo” or “shield”) and exploring action words connected to them that progress can be made. Second, we are mindful of the fact that at times, working with existing metaphors can even be harmful, especially when their literal or simplistic interpretations are taken up by patients or therapists. For example, Stilwell et al. (2021) notice that the use of linear structural metaphors (such as: pain is a “knot,” body is a “machine,” or one does not have “core stability”) may limit a person’s ability to understand their condition, as it suggests to the patient that the cause of their persistent pain is, e.g., “simply a muscle knot, rather than a complex experience” (p. 6). It is therefore important for the therapist to be sensitive to the effects a metaphor (and its co-construction) can have on a client. Third, while at times it is useful to explore the metaphor to its fullest, the therapist should also know when to let it go. The therapist should maximize the metaphor, and when it is no longer useful, let go of it, so that the client can focus on new experiences going further in life. One should not be “hooked” on the metaphors that are successful but see them as tools. Once they are used successfully (their meaning co-constructed with a client, allowing him/her to find a new perspective), it should be left aside, so that it does not constrain the client going forward.^16^

We can therefore further inquire about the role of metaphors in our proposal. If using action words is what brings about the relevant change, why do not we simply apply action words to any words, but metaphors? Could not we just focus on introducing many action words in a therapeutic dialog, or use action words on diagnostic words, without finding a metaphor first? What is special about enacting metaphors?

To provide an answer to this question, it is useful again to remember the embodied qualities of metaphors as discussed within the EEC perspective. Firstly, metaphors have embodied roots that can be explored. They afford talking about multi-sensorial aspects of our experiences (Abrahamson, 2020). Once the embodied roots and sensorial aspects of metaphors are discovered, it is easier to find action words to explore those metaphors further. Secondly, metaphors refer to a network of meanings. They can trigger associations made in different contexts and allow us to apply them to a present context. For instance, source objects of metaphors (harness and tattoo) are embedded in different practices and doings (literature, movies, or everyday life), which affords talking in new ways about the target: the therapist can tap into our shared experiences and cultural narratives to do with shields and tattoos to find relevant action words that can be used to further the conversation. Metaphors thereby help the therapist tap into the dialog, and find many action words, to deepen the conversational topic. Finally, some metaphors might work better than regular words, because they allow concrete sensorimotor visualizations. For instance, in a therapeutic dialog, a systemic therapist can invite his client to convert their statement into an image and draw the client’s attention to their implicit phenomenological modalities (somatic, proprioceptive, and kinesthetic).^17^ Take as an example the words “helpless” and “powerless” (Fondelli and Rucińska, 2021). While they seem interchangeable in a conversation, these words allow us to imagine being in different situations. Being helpless can be imagined as being without anyone around on whom one can rely (one is alone in the imagining). Being powerless can be imagined as not being able to influence someone who is there (one is not alone in the imagining). These imaginings, in turn, invite different action words. Thus, once one visualizes how the body is placed in those relations, new action words with new action possibilities can emerge. The enactive account to imagination, which proposes that imaginings are deeply rooted in bodily experiences and affects (Rucińska and Gallagher, 2021) can further clarify how new bodily experiences can come about with imagining.

To repeat, in our view, it is not what the metaphor says, but what one can do with the metaphor to bring about a sense of agency, that makes the metaphor work. As was our message all along, in different contexts and interactions, the meanings of metaphors can change with use.

## Data Availability Statement

The data analyzed in this study is subject to the following licenses/restrictions: The data presented in this study are available on request from the second author. The data are not publicly available due to privacy or ethical problem. Requests to access these datasets should be directed to TF, thomas.fondelli@telenet.be.

## Ethics Statement

Ethical review and approval was not required for the study on human participants in accordance with the local legislation and institutional requirements. Informed consent was obtained from the subjects involved in the therapeutic encounter and their legal guardian/next of kin for the publication of any potentially identifiable images or data included in this article.

## Author Contributions

All authors listed have made a substantial, direct, and intellectual contribution to the work and approved it for publication.

## Funding

This work was made possible thanks to the Research Foundation – Flanders (FWO) grant “Enactive Approach to Pretending” (12J0419N).

## Conflict of Interest

The authors declare that the research was conducted in the absence of any commercial or financial relationships that could be construed as a potential conflict of interest.

## Publisher’s Note

All claims expressed in this article are solely those of the authors and do not necessarily represent those of their affiliated organizations, or those of the publisher, the editors and the reviewers. Any product that may be evaluated in this article, or claim that may be made by its manufacturer, is not guaranteed or endorsed by the publisher.

## Footnotes

^1^Thanks to the anonymous reviewer for the helpful phrasing.

^2^For example, Jackson (1957) emphasized the importance of paying attention to the analogue aspects of communication, such as breath, tone of voice, and bodily movements; Satir et al. (1991) emphasized the importance of “experiencing” during therapeutic conversations and invited family members to sculpt their relationships through their bodies. And more recently, Wilson et al. (2020) emphasise how systemic therapy is first and foremost about enactment, or a performance, that is created between all the participants.

^3^Bertrando and Gilli (2008) note that there is a gap between, on the one hand, systemic theory’s focus on language and stories, and on the other hand, systemic practice with its attention to the effect these stories have on the bodily experiences of the client. They describe how the narrow focus on language and dialogue in systemic theory building and its “growing tendency (…) to emphasize words and narration, obscuring the relevance of body interaction” (Bertrando and Gilli, 2008, p. 368) has had an effect of creating the so-called “disembodied dialogues”: dialogues focused on the content of what is said that they underemphasize embodied experiences and non-verbal interactions.

^4^We note that while we think that our insights about metaphors are not just specific to systemic collaborative therapy and could be applied to other therapies, there are two main reasons why we stick to systemic collaborative therapy. One, the systemic therapeutic context is a better context for enacting metaphors, as in systemic therapy, we do not use metaphors to uncover meanings, but to create them. Two, enacting metaphors in typical talking therapies where the focus is still on “uncovering meanings” can yield opposite results than the ones we find beneficial from our perspective. We will return to this below.

^5^In this paper, we will not draw on the technical distinction between a metaphor, a simile, or an analogy. These distinctions are not relevant to make our argument: even if it turned out that some of our examples are not *stricte* about metaphors but should be linguistically classified as similes or analogies, our insights are just as relevant for therapeutic contexts.

^6^The context they consider is pretend playing that one is a meteor to learn about their trajectories and associated concepts from physics. They describe an experiment whereby children learned about trajectories of meteors by “embodying it” in pretense, such as by running and jumping, and explicit navigation of the space around them. In the context of the game, the children’s running had a metaphoric nature, as the running enacted the movement of the meteor. Pretend play involves a metaphoric transformation where one *acts-as-if* one thing is another, and it is precisely in this action that the banana (target) is a phone (source).

^7^For instance, being able to see similarities between two distinct objects is characterized by Norbury (2005) as “a complex developmental process that requires the acquisition of a number of skills [such as] broad enough semantic representations to capture the comparison being made” (p. 384). Norbury thinks that deciding in what respects two objects are similar involves thinking through the mappings from source to target domain with the use of “analogical reasoning skills” (2005, p. 385).

^8^“At the heart of metaphor is the direct pick-up of (resonance to) an invariant pattern in an informational array that is specific to both the topic and the vehicle (such as, for flowers and fireworks, invariants that specify progressive expansion from a central point). In this way, metaphor, as other forms of knowing, relies on a fundamentally perceptual process” (Szokolszky, 2006, p. 87).

^9^Such broad characterization of simulation does not commit one to Simulation Theory that is representational.

^10^As discussed by Kompa, “there are fMRI-studies that show that processing verbs, which denote actions performed by hand (pick, grasp), foot (kick) or mouth (lick) elicits activation in the motor (and premotor) cortex (in a somato-topically organized manner)” (2017, p. 197).

^11^For instance, a therapeutic context might require a different kind of metaphors than ones required for learning a skill; for the latter, see Abrahamson (2020).

^12^They give an example of making “long sweeping movements from the left to the right and back again” (p. 19) to enact the concept of a “bridge.”

^13^“These metaphors became enactive through clinician-patient interaction; we observed dynamic sense-making unfold between clinicians and patients when clinicians *touched patients* in the areas of their back where they were experiencing pain, as well as the surrounding areas. As the clinicians *applied manual pressure to tissues*, it was brought to patients’ attention when muscles were perceived by the clinician to be knotted, tight or ropey” (Stilwell et al., 2021, p. 239, emphasis added).

^14^Thanks to the anonymous reviewer for helpful wording.

^15^For instance, enacting metaphors in therapies where the focus is still on “uncovering meanings” can yield opposite results than the ones we find beneficial from this intervention method (which is bringing about the client’s sense of agency). For instance, it is possible to enact metaphors by focusing on action words connected to those metaphors to uncover more “hidden truths” about a client. One would do that by focusing on action words that deepen the original understanding of the metaphor (e.g., focusing on the heaviness of carrying a “harness”). But that would only reinforce the client in the old meanings (e.g., harness as a representation of the weight of bullying) and not yield new perspectives for the client, which ultimately, is the added value of our approach.

^16^Consider a useful analogy from Abrahamson (2020) on how a music teacher of cello uses a metaphor “grasp it like a strawberry” to convey what the learner’s grip should be like (gentle, as if one was afraid to squish a strawberry). Once the student learns the grip to find for oneself a musical flow, the metaphor has served its purpose and should be let go. The student should no longer think of the metaphor of a strawberry when playing, but focus on the new musical experiences, and affordances for playing, that the mastering of the grip has allowed.

^17^According to Abrahamson (2020), metaphor understanding, and capacity to learn from metaphors lies in the emergence of “multiple sensory modalities tacitly springing forth [in the enactment of the metaphor], including the visual, auditory, kinesthetic, proprioceptive, and somatic” (p. 216).
